# Supplementary material for: COZOID: contact zone identifier for visual analysis of protein-protein interactions
Source: BMC Bioinformatics. 2018 Apr 6;19:125. doi: 10.1186/s12859-018-2113-6 (PMC5889581; doi:10.1186/s12859-018-2113-6)
Supplement: Supplementary file 2 — Software build. Executable binary file of the software tool. (ZIP 118,784 kb) [file 12859_2018_2113_MOESM2_ESM.zip › cozoid/platform/docs/testng-6.5.1-javadoc.zip!/org/testng/package-summary.html]

org.testng (TestNG)


---


|  |  |  |
| --- | --- | --- |
| **Overview** \| **Package** \| Class \| **Tree** \| **Deprecated** \| **Index** \| **Help** | |  |
| PREV PACKAGE   **NEXT PACKAGE** | **FRAMES**    **NO FRAMES**     **All Classes** |  |


---

## Package org.testng

| **Interface Summary** | |
| --- | --- |
| **IAnnotationTransformer** |  |
| **IAnnotationTransformer2** | Use this interface instead of IAnnotationTransformer if you want to modify any TestNG annotation besides @Test. |
| **IAttributes** | A trait that is used by all interfaces that lets the user add or remove their own attributes. |
| **IClass** | `IClass` represents a test class and a collection of its instances. |
| **IConfigurable** | If a test class implements this interface, its run() method will be invoked instead of each configuration method found. |
| **IConfigurationListener** | Listener interface for events related to configuration methods. |
| **IConfigurationListener2** |  |
| **IConfigureCallBack** | A parameter of this type will be passed to the run() method of a IConfigurable. |
| **IExecutionListener** | A listener used to monitor when a TestNG run starts and ends. |
| **IExtraOutput** | This class is used by Reporter to store the extra output to be later included in the HTML report: - User-generated report - Parameter info Created on Feb 16, 2006 |
| **IHookable** | If a test class implements this interface, its run() method will be invoked instead of each @Test method found. |
| **IHookCallBack** | A parameter of this type will be passed to the run() method of a IHookable. |
| **IInstanceInfo** | This class defines a pair of instance/class. |
| **IInvokedMethod** | An interface representing a method that has been invoked by TestNG. |
| **IInvokedMethodListener** | A listener that gets invoked before and after a method is invoked by TestNG. |
| **IInvokedMethodListener2** | Implement this interface if you need a handle to `ITestContext`. |
| **IMethodInstance** | This interface captures a test method along with all the instances it should be run on. |
| **IMethodInterceptor** | This class is used to alter the list of test methods that TestNG is about to run. |
| **IMethodSelector** | This interface is used to augment or replace TestNG's algorithm to decide whether a test method should be included in a test run. |
| **IMethodSelectorContext** | An implementation of this interface is passed to all the Method Selectors when their includeMethod() is invoked. |
| **IModuleFactory** | This interface is used by the moduleFactory attribute of the @Guice annotation. |
| **IObjectFactory** | Factory used to create all test instances. |
| **IObjectFactory2** | Factory used to create all test instances. |
| **IReporter** | This interface can be implemented by clients to generate a report. |
| **IResultMap** |  |
| **IRetryAnalyzer** | Interface to implement to be able to have a chance to retry a failed test. |
| **ISuite** | Interface defining a Test Suite. |
| **ISuiteListener** | Listener for test suites. |
| **ISuiteResult** | This class represents the result of a suite run. |
| **ITest** | If a test class implements this interface, it will receive a special treatment, such as having the test name displayed in the HTML reports. |
| **ITestClass** | This class represents a test class: The test methods The configuration methods (test and method) The class file Note that the methods returned by instances of this class are expected to be correct at runtime. |
| **ITestClassFinder** | This class is used by TestNG to locate the test classes. |
| **ITestContext** | This class defines a test context which contains all the information for a given test run. |
| **ITestListener** | A listener for test running. |
| **ITestMethodFinder** | This interface allows to modify the strategy used by TestRunner to find its test methods. |
| **ITestNGListener** | This is a marker interface for all objects that can be passed as a -listener argument. |
| **ITestNGListenerFactory** | A factory used to create instances of ITestNGListener. |
| **ITestNGMethod** | Describes a TestNG annotated method and the instance on which it will be invoked. |
| **ITestObjectFactory** | Parent interface of all the object factories. |
| **ITestResult** | This class describes the result of a test. |
| **ITestRunnerFactory** | A factory for TestRunners to be used by SuiteRunners. |

| **Class Summary** | |
| --- | --- |
| **Assert** | Assertion tool class. |
| **AssertJUnit** | A set of assert methods. |
| **ClassMethodMap** | This class maintains a map of `>`. |
| **CommandLineArgs** |  |
| **ConversionUtils** | Helper methods used by the Eclipse plug-in when converting tests from JUnit. |
| **Converter** | Convert XML files to YAML and vice versa. |
| **DependencyMap** | Helper class to keep track of dependencies. |
| **FileAssert** | Assertion tool for File centric assertions. |
| **Reporter** | This class is used for test methods to log messages that will be included in the HTML reports generated by TestNG. |
| **ReporterConfig** | Stores the information regarding the configuration of a pluggable report listener. |
| **ReporterConfig.Property** |  |
| **SuiteRunner** | `SuiteRunner` is responsible for running all the tests included in one suite. |
| **SuiteRunnerWorker** | An `IWorker` that is used to encapsulate and run Suite Runners |
| **SuiteRunState** | A state object that records the status of the suite run. |
| **TestListenerAdapter** | A simple ITestListener adapter that stores all the tests that were run. |
| **TestNG** | This class is the main entry point for running tests in the TestNG framework. |
| **TestNG.ExitCodeListener** |  |
| **TestNGAntTask** | TestNG settings: classfileset (inner) classfilesetref (attribute) xmlfileset (inner) xmlfilesetref (attribute) enableAssert (attribute) excludedGroups (attribute) groups (attribute) junit (attribute) listener (attribute) outputdir (attribute) parallel (attribute) reporter (attribute) sourcedir (attribute) sourcedirref (attribute) suitename (attribute) suiterunnerclass (attribute) target (attribute) testjar (attribute) testname (attribute) threadcount (attribute) dataproviderthreadcount (attribute) verbose (attribute) testrunfactory (attribute) configFailurepolicy (attribute) randomizeSuites (attribute) methodselectors (attribute) Ant settings: classpath (inner) classpathref (attribute) jvm (attribute) workingDir (attribute) env (inner) sysproperty (inner) propertyset (inner) jvmarg (inner) timeout (attribute) haltonfailure (attribute) onHaltTarget (attribute) failureProperty (attribute) haltonFSP (attribute) FSPproperty (attribute) haltonskipped (attribute) skippedProperty (attribute) testRunnerFactory (attribute) Debug information: dumpCommand (boolean) dumpEnv (boolean) dumpSys (boolean) |
| **TestNGAntTask.TestNGLogSH** |  |
| **TestNGUtils** |  |
| **TestRunner** | This class takes care of running one Test. |

| **Enum Summary** | |
| --- | --- |
| **TestNGAntTask.Mode** |  |

| **Exception Summary** | |
| --- | --- |
| **SkipException** | The root exception for special skip handling. |
| **TestException** | Exception thrown when an exception happens while running a test method. |
| **TestNGException** | The base class for all exceptions thrown by TestNG. |
| **TimeBombSkipException** | A `SkipException` extension that transforms a skipped method into a failed method based on a time trigger. |

---


|  |  |  |
| --- | --- | --- |
| **Overview** \| **Package** \| Class \| **Tree** \| **Deprecated** \| **Index** \| **Help** | |  |
| PREV PACKAGE   **NEXT PACKAGE** | **FRAMES**    **NO FRAMES**     **All Classes** |  |


---
